# A process for instituting best practice in the intensive care unit

**DOI:** 10.4103/0972-5229.42562

**Published:** 2008

**Authors:** Elisabeth L. George, Patricia Tuite

**Affiliations:** **From:** University of Pittsburgh Medical Center Presbyterian, Pittsburgh, PA, USA; 1 University of Pittsburgh, School of Nursing, Pittsburgh, PA, USA

**Keywords:** Evidence based practice, quality, multi-disciplinary teams, critical care nursing

## Abstract

Goals of health care are patient safety and quality patient outcomes. Evidence based practice (EBP) is viewed as a tool to achieve these goals. Health care providers strive to base practice on evidence, but the literature identifies numerous challenges to implementing and sustaining EBP in nursing. An initial focus is developing an organizational culture that supports the process for nursing and EBP. An innovative strategy to promote a culture of EBP was implemented in a tertiary center with 152 critical care beds and numerous specialty units with diverse patient populations. A multi-disciplinary committee was developed with the goal to use evidence to improve the care in the critical care population. EBP projects were identified from a literature review. This innovative approach resulted in improved patient outcomes and also provided a method to educate staff on EBP. The committee members have become advocates for EBP and serve as innovators for change to incorporate evidence into decision making for patient care on their units.

In 2000, the Institute of Medicine released a number of reports focusing on the safety of health care in the United States that were very pertinent to physicians and nurses. The most famous statement from one of the reports stated that the number of medical errors committed in hospitals resulted in 44,000 to 98,000 deaths per year.[1] That statement resulted in many lay persons taking significant interest in the care that was being provided within the hospitals. Thus, most health care professionals began to critically assess medical treatment.

## Quality Care and Evidence-Based Practice

Multidisciplinary teams have begun to look at methods to improve the quality of care administered within the healthcare system. The Institute of Medicine defined the quality of healthcare as care that is safe, timely, effective, efficient, equitable, and patient-centered.[2] This definition has been the focus when developing programs aimed at improving the quality of care within organizations. A variety of measures have been used to guide quality improvement initiatives such as those focusing on structural measures, process measures, or outcome measures.[3]

Structural measures include those that reflect the setting or system where care is delivered or the way that care is organized. Examples of structural measures include number of procedures completed, the number of trained surgeons within the organization,[4] the size of an intensive care unit (ICU), whether or not the unit is open or closed, or the amount of technology available.[4] Processes describe the care that the patient actually receives or analyzes what we do as practitioners.[3,4] Examples of process measures applicable to an intensive care unit (ICU) include daily interruption of sedation, deep vein thrombosis prevention, or daily spontaneous breathing trials. Finally, measuring outcomes is the final way to assess quality and refers to the results that are achieved. Outcomes most frequently measured include: morbidity, mortality, length of stay and readmission rates. Other frequently measured variables for the ICU population include the number of ventilator associated pneumonias or central-line bloodstream infections.

Evidence-based practice (EBP) is a problem solving approach by which the healthcare provider makes clinical decisions using the best available scientific evidence, one′s clinical experiences, and patient preferences in the context of available resources.[5] In order to provide the best quality, the health care team should make decisions based upon best practice. This may require working with the team to evaluate the level of evidence and then a critical appraisal of the evidence to establish best practice. The Agency for Healthcare Research and Quality has established a hierarchy for the levels of evidence [Table 1]. This hierarchy can be very beneficial when reviewing the literature and determining which studies to evaluate.

**Table 1:** Strength of evidence to support practice.[8]

Level I	Meta analysis of multiple studies
Level II	Experimental studies
Level III	Well developed quasi-experimental studies
Level IV	Well developed non-experimental studies
Level V	Case reports and clinical examples

EBP is viewed as an avenue to provide the high quality of care we strive for. EBP adds evidence to our daily clinical care decisions in addition to our standard sources for decision making: tradition and our experience. In order to implement evidence-based practice changes and improve the quality of the care provided within the organization, the group must decide on both the practices for change and the model to guide this process. The best way to integrate this process into practice is through the development of a committee to facilitate evidence-based practice and quality improvement.

This article addresses one example of the structure, process, and outcomes of a nurse led committee that has been successful at incorporating these principles into the critical care environment. Within our current practice setting, this was accomplished through the development of the "Rules of Evidence Committee". This committee laid the foundation for the implementation of quality improvement projects, policy and procedure changes based on current evidence, and the facilitation of bedside process changes through physician and nursing rounds.

## Structure

The hospital is a 834 bed teaching hospital with nine intensive care units (ICU) and approximately 152 ICU beds. Each ICU has a critical care physician as medical director and a nursing unit director. There is also a clinical nurse specialist (CNS) assigned to cover the ICUs. A CNS is a nurse educated with a master′s degree in nursing. The CNS′s practice focuses on the care of patients and their families, supporting other healthcare workers, and working at a system′s level to enact change. The CNS serves as an expert practitioner, educator, consultant, facilitator of research, leader, and mentor. At this hospital, the clinical nurse specialist (CNS) was charged with providing high quality, cost-effective care within this environment, and began to look for projects to meet this goal.

Rather serendipitously, our committee formed in 2002. While the CNS was working on educating the nursing staff on prevention of ventilator associated pneumonia (VAP), and creating a head of bed (HOB) protocol, several of the critical care fellows doing a quality improvement rotation had plans to work on the same project. This resulted in nursing and critical medicine joining efforts. This leadership structure has had a direct impact on the productivity and sustainability of the committee.

The first goal of the committee was to focus on VAP prevention by implementing a HOB standard in our intensive care units (ICUs). A multidisciplinary team was established to accomplish these efforts. Members were selected based on their role or on the unit were they worked. Our goal was to have a staff nurse representative from each ICU. This ensured representation of the rather heterogeneous population of patients in our 9 ICUs. The nursing members were either selected by the unit nursing director or volunteered.

The multidisciplinary committee membership was comprised of nursing staff representatives, critical care physicians, infection control practitioners, respiratory therapists, physical therapists, maintenance staff, radiology, and enterostomal therapy nurses.

It is important to note that nursing administration support was included. Administration reinforcement is the key to success in ensuring that the staff attend meetings and for the implementation of projects. In addition, a nursing faculty member from a local university partnered with the group to assist with data collection and analysis; as well as educational support for staff. Some members are self explanatory such as the need for respiratory therapy to improve the VAP rates, but one may question maintenance. The maintenance department did a quality check for all ICU beds to ensure that an accurate HOB angle could be read on beds for proper positioning.

The group′s goal was altered to be more encompassing than VAP prevention. Our committee goal was “to improve the care in our critical care population by applying standards of best practice to the daily care of our patients”. The title for the group was the Rules of Evidence committee. A rule of evidence (ROE) is an aspect of care provided to critically ill patient that should be done by all practitioners everyday. A ROE is based upon evidence and when compared to usual practice, patient care outcomes are superior.

The group meets monthly at a consistent day and time based upon group consensus. Agendas are distributed prior to the meetings so the group can prepare. Meetings are about 1- 1.5 hours and staffs are compensated for the time.

## Process

Initially, it was important for the CNS and the critical care physician to provide leadership during the first project. This allowed them to lead by example and demonstrate the skills necessary to facilitate change. The CNS explained each step to the committee representatives to build their skill and confidence in EBP. The overall plan was to progressively increase independent work and accountability of the staff nurse as knowledge and experience increased. Phase one would be CNS lead, phase two would increase the committee nurse representative involvement and develop the staff nurses as ROE leader of their unit and phase three would progress to the staff nurse identifying and leading projects. If each ICU did at least one project per year, the ICU team would have nine project outcomes to implement within the other ICU units.

### Phase one

The VAP initiative was the first CNS led project. To develop the EBP knowledge of the nursing staff, the project was structured to follow an EBP model. The model chosen was the Rosswurm and Larrabee model that encompasses the following steps: 1) Assess the need for a practice change, 2) Link problem, intervention, and outcomes, 3) Synthesize best evidence, 4) Design the practice change, 5) Implement and evaluate practice change, and 6) Integrate and Maintain practice change.[6]

The first step was assessing the need for a change and at the time VAP was already identified as crucial component of ICU care because of the reported high incidence of hospital acquired pneumonias. To identify the significance of VAP and to help the staff to understand why VAP was targeted, the VAP statistics related to increase in mortality, length of stay, and cost were presented at a meeting. Then literature that supported the practice change was identified. Several studies were located including one randomized trial.[7]

The CNS presented this literature to the committee members so they knew the rationale for a practice change. In addition, this exercise was also useful to demonstrate the process of searching and synthesizing literature. Based on the literature, development of a HOB protocol began.

Armed with the literature, the QI fellows audited the ICU units checking the HOB elevation during all days and various shifts. Current unit specific data was then available to convey to the staff that current practice did not demonstrate current standards. Overall, in the ICUs patients HOB was greater than 30° less than 30% of the time.

Step 4 or designing the change was very time consuming. Setting outcomes for decreasing VAP, mortality, and ICU LOS was easy. Developing this process took over 9 months, because at the time there were 10 medical directors that had to agree on the HOB protocol. This is when the physician leadership was crucial. The ICU physician leader served as the liaison between the committee working to develop the protocol and the ICU medical directors. Some physicians agreed to a standard of HOB 30°, but others felt the standard should be 45° since that is the angle used in a reported randomized study comparing HOB elevation and VAP. ROE committee member were updated on progress and issues throughout this process to facilitate their education in the EBP process.

The leadership also learned throughout this process. Our goal was to develop the perfect protocol prior to implementation, but this was not achieved. Once a general consensus on the protocol was reached, it was decide to proceed with the implementation step and to be prepared to make alterations as needed. The implementation step of the process proved to be the most difficult.

As with any practice, education is viewed as the first step. We utilized a VAP self learning module that all ICU nurses and respiratory therapist had to complete. In addition, a modified version of the VAP education model was administered to physical therapists and radiology technicians. A poster identifying the evidenced based focus for HOB elevation to prevent VAP and information on the HOB protocols was developed and displayed in all the ICUs. One physician even gave an idea for a logo and sign to be posted at patient bedsides to remind staff of the initiative. To make the protocol accessible, copies of the protocol were also posted at each bedside where the nurse routinely performs documentation. In addition, unit based educational sessions were provided on the initiative. To coincide with the implementation, maintenance did their quality checks of the ICU beds.

During the initial period, the CNS did weekly rounds on all ICU beds to track compliance with the HOB initiative as well as to provide individualized education to staff and to answer questions. The rounds also provided data that facilitated timely feedback on compliance to the units. Feedback was provided monthly with graphs identifying unit HOB compliance compared to unit VAP rates. The graphs were posted on the units and served as good teaching tools for the staff.

Overtime, compliance increased from the initial 30% to 70% with the HOB elevation protocol. This success was shared with staff via an ICU newsletter and messages were sent to staff via electronic mail. Still not satisfied with this outcome, an additional implementation strategy was instituted via the hospital electronic medical record. A space was designated on the vital sign sheet for the nurse to chart the patient′s HOB and any exceptions to the standard when obtaining vital signs. At this same time, the HOB protocol was made available in the electronic charting as a reference.

The rounds to assess compliance were positive as staff associated the CNS with HOB often hustling to get patients HOB elevated when the CNS was on the unit. On the other hand, the physician leader was concerned about the time required to complete this work and suggested utilizing the documented HOB that was built into our electronic documentation system. To determine if charting of HOB matched reality, we compared the results of the two assessments. Initial results indicated only a 31% average agreement between what was charted and the actual position of the patient. Since initial adoption of the HOB protocol, unit rounds have decreased to one time per week and additional auditors have been added so that staff does not associate just one person with the HOB initiative.

Throughout this work, it was determined that VAP is multifaceted and other factors that impact VAP were addressed, such as standardizing mouth care, use of sterile water for mouth care, chlorhexidine rinse (in select patient populations), and checking endotracheal cuff pressures. The VAP initiative continues in this institution. The percentage of VAP rates continues to be tracked and evaluation of the process is ongoing.

### Phase two

After completing our demonstration project, we started a phase two project, with the goal to incorporate the staff nurse member more in the project work and to build the nurse′s self confidence and skill in the EBP process. To prepare the nurses for this next phase, the leaders took the opportunity to educate staff members on the EBP Process, and developing questions for inquiry. Classes were held at the onsite library to provide skills required to search the literature. Education was also provided on methods for data collection. The nursing faculty assisted with teaching strategies on critically appraising the literature. It was also determined that it was important to share with the group how topics were selected as projects to meet our goals of improving care to our ICU patients. Sessions were held to discuss what qualifies as a good EBP project, noting that it should impact a critical component of patient care such as mortality, quality and costs. While we did not formally audit the group, members gave positive comments about the opportunities to learn.

Daily interruption of sedation was the project selected for phase II. This process intervention has been associated with VAP rates so it seemed an ideal second project.

The same EBP process was utilized but the staff was more engaged. For example, some staff nurses who volunteered participated in the protocol development and were able to experience the tedious process required to gain consensus among the ICU medical directors. Collaborative work with the pharmacist was also necessary to achieve this work. The protocol needed to be approved by numerous committees including: the policy and procedure committee and pharmacy and therapeutics. The staff nurses attended these meetings and were exposed to the steps required to facilitate change. Other staff assisted with the data collection, pre and post intervention. The process measures continue to be tracked.

The biggest change associated with phase two was having the committee representatives disseminate the protocol and provide the education to their units. The CNS developed an educational PowerPoint with their input for this purpose. Utilizing knowledge from change theory, we again used multiple modes of education to disseminate information such as, posters and the ICU newsletter. This method provided a way for the ROE committee representative to be seen as the unit champion on EBP. The unit staff saw the representative as the “expert” person available to answer questions. We hypothesized that there may be value in hearing it from “one of your own” instead of the CNS.

### Phase three

In phase three of our committee, the focus shifted to the representatives from each unit and they were asked: “What aspect of patient care on your unit most needs to be improved?” Our first member led project was a CNS student of our nursing faculty so that helped to ensure the project was completed. It was a great example for the committee to demonstrate that a nurse could complete the process with success. Examples of representative led projects completed or ongoing include: central line tubing change frequency, family presence with physician rounds and/or emergencies, blood conservation methods, and adjustment of monitor alarms to reduce alarm false error rate to increase nurse sensitivity to alarms. This phase remains very difficult to engage the staff nurse to take on the responsibility.

## Lessons Learned

The leadership work for this process is time consuming and requires preparation for meetings, assisting representatives with work in between meetings, providing feedback to the units, and facilitating the multidisciplinary involvement in each project.

Strategies that have promoted success include the support of nursing administration to encourage nurse attendance, the multi-disciplinary approach to involve all experts, the nursing faculty collaboration to facilitate data collection/analysis, and the shared leadership between nursing and critical care medicine to maintain the committee′s initial goal. The committee structure shifted some of decision making from the hospital leaders to the individuals who carry out the day-to-day care and it was well received by the nurses.

The most important factor contributing to the committee′s success is the collaboration between the CNS and physician leader. The physician attends all meetings and this sends a strong message to the group that the work is important. In addition, the physician is always available to the CNS for guidance.

Membership changes to our group have often slowed progress on some projects, but in some instances new membership has contributed to fresh insight. Therefore, we are planning to set a time limit for membership to two years, as this format will allow the committee to benefit from new membership as well as develop more "champions" in EBP and quality for the ICUs. The reality is that in some months there is low attendance at monthly meetings which also impacts productivity.

Over time we have gone back and forth between implementing a process idea in one ICU then rolling out to others *vs* rolling out the process change to all ICUs at once. There is not one fail-proof approach; the choice of approach is dependent on the topic.

Challenges to implementation exist at each stage of the process due to general opposition to change practice from multiple areas, the diversity of specialties in our nine ICUs and the work that is required to reach consensus from multiple disciplines. The leadership worked to overcome these barriers by sustaining the work of the project and utilizing a consistent approach to project development.

Focusing on compliance measures and outcomes is an important component of process change, but it can be very time consuming for the CNS. To overcome this barrier, other CNSs and respiratory therapists have collected data and the nursing faculty assists with data management. Alternative ideas currently being investigated include electronic devices for data collection and involving the committee members to collect outcome data in their ICU.

Emphasizing the committee′s success is essential to provide momentum. Nursing members have presented projects at local and national conferences.

## Conclusion

It is essential that we focus on providing the best possible care for patients. Healthcare providers can ensure that high quality care is provided by ensuring that a process is in place to monitor care. It is also essential that the care provided is based upon sound scientific evidence. Forming a multidisciplinary committee whose focus is on quality as well as utilizing best evidence is one way to accomplish this goal. In conclusion, we found that developing a committee with key leadership to provide direction to the group to prioritize projects, implement change and measure performance resulted in success. In addition, our multidisciplinary team with a strong nursing and physician leadership also created the opportunity to engage staff nurses in EBP knowledge and to improve the care of patients in our ICUs.
